# Supplementary material for: Furanic Humins from Biorefinery as Biobased Binder for Bitumen
Source: Polymers (Basel). 2022 Mar 3;14(5):1019. doi: 10.3390/polym14051019 (PMC8912838; doi:10.3390/polym14051019)
Supplement: Supplementary file 1 [file polymers-14-01019-s001.zip › polymers-1556552-supplementary.pdf]

Supplementary Materials:

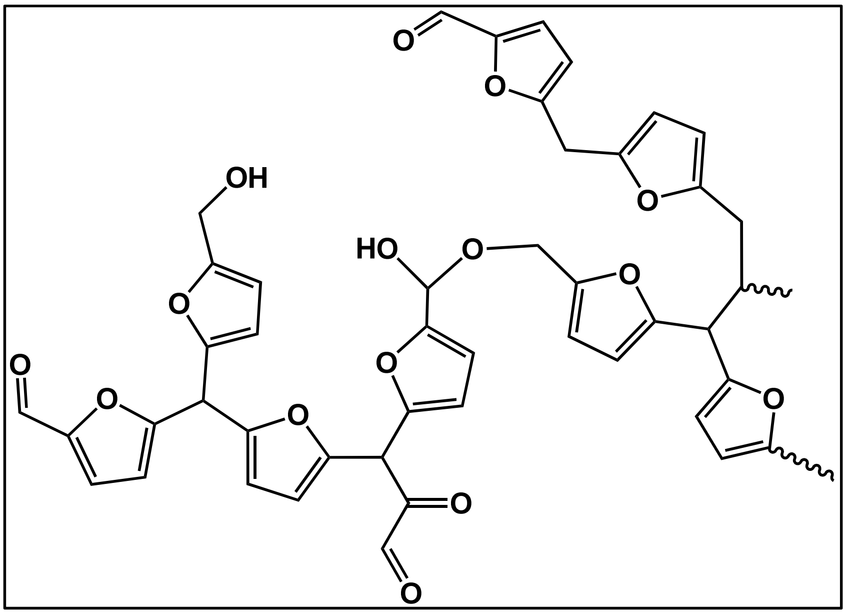

Figure S1. Representative structure of furanic humins employed in this study.

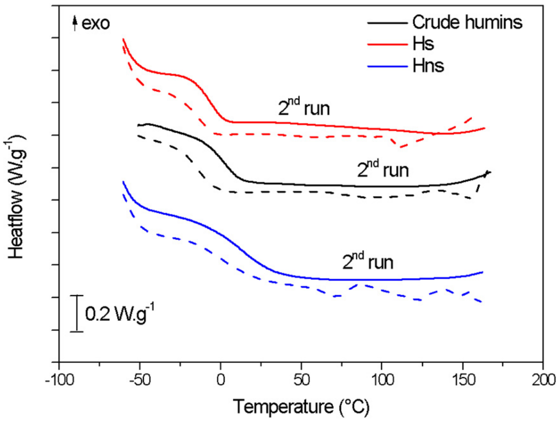

Figure S2. DSC scans from Hs (red lines) and Hns (blue lines), compared with crude humins sample (black lines) at heating rates of 30 °C.min^{-1}. The first heating scan is indicated by dash lines and the second heating scan by solid lines.

Table S1. T_g value of crude humins Hs and Hns at 30°C.min^{-1} obtained from the 1st and the 2nd DSC scans.

	T_g/1st scan	T_g/2nd scan
Crude humins	−14 °C	2 °C
Hs	−17 °C	−6 °C
Hns	0 °C	10 °C
